# A Comprehensive Assessment of Genetic and Epigenetic Alterations Identifies Frequent Variations Impacting Six Prototypic SCF Complex Members

**DOI:** 10.3390/ijms23010084

**Published:** 2021-12-22

**Authors:** Rubi Campos Gudiño, Ally C. Farrell, Nicole M. Neudorf, Kirk J. McManus

**Affiliations:** 1 CancerCare Manitoba Research Institute, CancerCare Manitoba, Winnipeg, MB R3E 0V9, Canada; camposgr@myumanitoba.ca (R.C.G.); farrella@myumanitoba.ca (A.C.F.); neudorfn@myumanitoba.ca (N.M.N.); 2 Department of Biochemistry & Medical Genetics, University of Manitoba, Winnipeg, MB R3E 0J9, Canada

**Keywords:** SKP1, CUL1, RBX1, SKP2, FBXW7, FBXO5, SCF complex, genome instability, chromosome instability, cancer

## Abstract

The SKP1, CUL1, F-box protein (SCF) complex represents a family of 69 E3 ubiquitin ligases that poly-ubiquitinate protein substrates marking them for proteolytic degradation via the 26S proteasome. Established SCF complex targets include transcription factors, oncoproteins and tumor suppressors that modulate cell cycle activity and mitotic fidelity. Accordingly, genetic and epigenetic alterations involving SCF complex member genes are expected to adversely impact target regulation and contribute to disease etiology. To gain novel insight into cancer pathogenesis, we determined the prevalence of genetic and epigenetic alterations in six prototypic SCF complex member genes (*SKP1*, *CUL1*, *RBX1*, *SKP2*, *FBXW7* and *FBXO5*) from patient datasets extracted from The Cancer Genome Atlas (TCGA). Collectively, ~45% of observed SCF complex member mutations are predicted to impact complex structure and/or function in 10 solid tumor types. In addition, the distribution of encoded alterations suggest SCF complex members may exhibit either tumor suppressor or oncogenic mutational profiles in a cancer type dependent manner. Further bioinformatic analyses reveal the potential functional implications of encoded alterations arising from missense mutations by examining predicted deleterious mutations with available crystal structures. The SCF complex also exhibits frequent copy number alterations in a variety of cancer types that generally correspond with mRNA expression levels. Finally, we note that SCF complex member genes are differentially methylated across cancer types, which may effectively phenocopy gene copy number alterations. Collectively, these data show that SCF complex member genes are frequently altered at the genetic and epigenetic levels in many cancer types, which will adversely impact the normal targeting and timely destruction of protein substrates, which may contribute to the development and progression of an extensive array of cancer types.

## 1. Introduction

In 2020, ~20 million individuals throughout the world were newly diagnosed with cancer, while ~10 million succumbed to the disease [1]. Despite these statistics, the molecular determinants (i.e., aberrant genes, proteins and pathways) underlying cancer development and progression remain poorly understood. Accordingly, new insight into the molecular events driving oncogenesis is needed to aid in the development of novel therapeutic strategies aimed at ultimately improving the lives and outcomes of those living with cancer. Decades of biochemical and genetic data have shown that aberrant expression/abundance of key proteins involved in critical biological processes (e.g., cell cycle regulation, DNA damage repair, and apoptosis [2,3,4,5,6,7,8]) are drivers of disease development and progression [9,10,11,12]. In this regard, the ubiquitin-proteasome system, an essential protein degradation system in eukaryotes, is a fundamental regulator of cell function at the protein level and its aberrant assembly and function are associated with malignant transformation and disease progression [13,14,15,16,17]. More specifically, the aberrant expression and function of the SCF (SKP1, S-phase Kinase associated Protein 1; CUL1, Cullin 1; F-box protein) complex occurs in an extensive array of cancer types [9,10,12,18,19,20]. However, additional studies are required to understand its role in disease pathogenesis as the molecular mechanisms underlying aberrant SCF complex function remain largely unknown.

The ubiquitin-proteasome system is best understood for its role in regulating protein abundance, where polyubiquitin chains are covalently attached to protein substrates through the activities of the E1 (ubiquitin activating), E2 (ubiquitin conjugating), and E3 (ubiquitin ligase) enzymes, targeting them for degradation by the 26S proteasome (reviewed in [13,21,22]). Ubiquitin ligation to a specific protein substrate requires recognition and binding by the E3 ligase enzyme [13,23], and so it is primarily responsible for conferring substrate specificity and ultimately, proteasomal degradation [24]. The SCF complex comprises the largest group of E3 ubiquitin ligases [25] that encompasses a diverse array substrate specificities that includes cell cycle regulators (e.g., Cyclin E1, P27) [2,18], transcription factors (e.g., c-MYC) [7,26] and regulators of DNA damage-response (e.g., RAD51) [3]. The SCF complex is comprised of three invariable core subunits, namely SKP1, CUL1 and RBX1 (Really Interesting New Gene [RING]-Box protein 1), and one of 69 variable F-box proteins conferring substrate specificity [4]. Conceptually, RBX1 interacts with the E2 enzyme and CUL1, a scaffolding protein that brings the F-box protein and its ligand within close spatial proximity of the E2 enzyme harboring the ubiquitin moiety to be transferred to the substrate protein upon binding with SKP1 [27,28]. In this regard, SKP1 serves as an adaptor between the SCF complex and the F-box proteins via its conserved F-box motif [29,30,31]. F-box proteins are classified into one of three families based on the presence (or absence) of specific protein motifs, including: 1) leucine-rich repeat (FBXL), 2) WD-40 (FBXW) and 3) other (FBXO) [27,30,32,33]. As there are 69 distinct F-box proteins there are 69 unique SCF complexes, each with distinct target specificities that are collectively proposed to regulate hundreds to thousands of protein substrates for ubiquitination [34]. Thus, aberrant expression of individual SCF complex members driven by genetic and epigenetic changes are expected to adversely impact an extensive array of proteins, which in turn is predicted to adversely impact biological pathways expected to contribute to the development and progression of cancer. Indeed, alterations in various SCF complex members occur in a vast array of cancer types and their altered expression has more recently been shown to promote various forms of genome instability and corresponds with early etiological events including cellular transformation [9,10,11,12] (reviewed in [20]).

In this study, we assessed both genetic and epigenetic changes in six key SCF complex member genes including the three invariable core members (i.e., *SKP1*, *CUL1* and *RBX1*) and a single representative member from each of the three F-box families, including *SKP2* (*FBXL1*), *FBXW7* (*CDC4*) and *FBXO5* (*EMI1*). Importantly, *SKP2* and *FBXW7* encode well studied F-box proteins and prototypical examples of an oncogene and tumor suppressor gene, respectively, while *FBXO5* encodes an F-box protein whose substrate specificities and role in disease development is poorly understood and requires further in-depth study. Using publicly available, patient-derived data from The Cancer Genome Atlas (TCGA) extracted from numerous cancer types [35,36,37], we assessed the prevalence and types of genetic and epigenetic alterations (gene mutations, copy number alterations [CNAs] and methylation status) within each gene to determine their potential implications in disease pathology. Overall, we determined that the six SCF complex member genes are frequently altered in solid tumor samples and predominantly harbor missense mutations predicted to be damaging. Overall, the mutations are generally distributed across the entirety of their coding sequences, with *FBXW7* exhibiting several alteration hot-spots. When combined with tertiary and quaternary structure (crystallographic) data, many of the encoded amino acid substitutions are predicted to adversely impact protein–protein interactions occurring between individual SCF complex members and/or their protein substrates, which is expected to prevent substrate protein degradation leading to their aberrant accumulation. Beyond mutations, we also discovered that CNAs occur frequently for all six genes and that while *CUL1* and *SKP2* predominantly exhibit copy number gains, the remaining genes (*SKP1*, *RBX1*, *FBXW7* and *FBXO5*) exhibit more copy number losses that correspond with reduced mRNA expression. Collectively, these data suggest that SCF complex members may act as oncogenes and/or tumor suppressor genes in a context-dependent or cancer-specific manner. Finally, we examined the methylation status of all six genes and determined that each is differentially methylated across cancer types, suggesting epigenetic misregulation may also contribute to cancer pathogenesis in a manner like that observed following gene copy number losses. Collectively, these findings provide novel insight into genetic and epigenetic alterations affecting the SCF complex and support the possibility that aberrant expression and/or function of key SCF complex members may be an early etiological event driving the development and progression of many cancer types.

## 2. Results

### 2.1. Genes Encoding SCF Complex Members Are Mutated Frequently in Cancer

**Figure 1:**
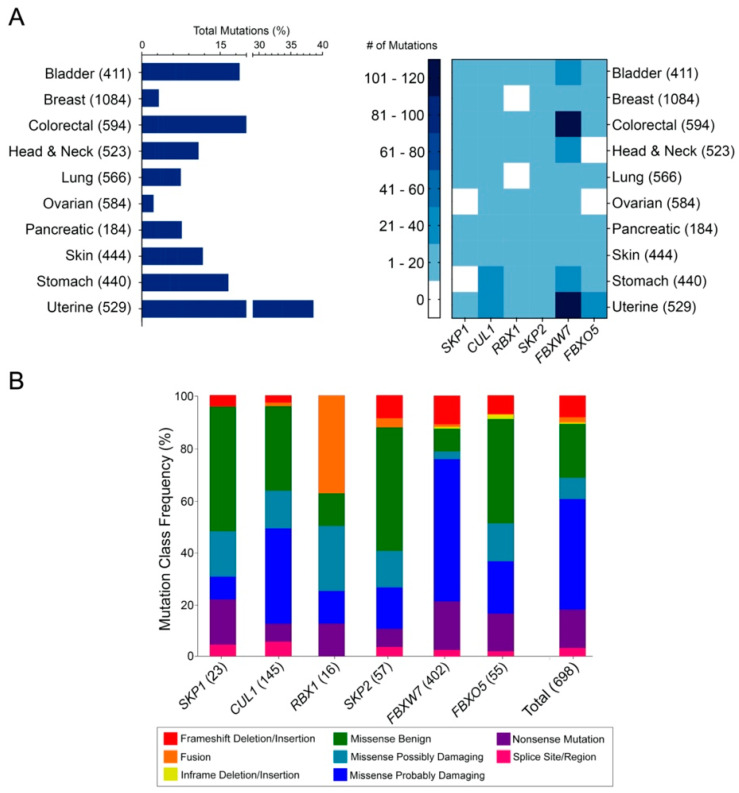
Frequency and types of mutations of six SCF complex member genes in 10 cancer types. (**A**) Bar graph (left) and heatmap (right) displaying the frequency and prevalence, respectively, of SCF complex member gene mutations in 10 cancer types with the total number of cancer cases indicated within brackets. Alteration categories include frameshifts, fusions, in-frame deletions/insertions, missense, nonsense and splice site mutations. (**B**) Bar graph presenting the frequency of the alteration categories from 10 cancer types, with the aggregate total frequencies provided in the last column (Total). The total number of alterations identified for each gene are indicated within brackets.

To determine the mutational frequency and types of alterations occurring in the six prototypic and representative SCF complex member genes (*SKP1*, *CUL1*, *RBX1*, *SKP2*, *FBXW7* and *FBXO5*) across various cancers, TCGA data [35,36,37] were assessed as detailed in Materials and Methods. Briefly, mutational data, including frameshift deletions/insertions, fusions, in-frame deletions/insertions, missense mutations (benign and damaging), nonsense mutations and splice site/regions mutations were extracted from 10 solid tumor types, and analyzed (Figure 1A), with the respective mutational frequencies provided in Table S1. As shown in Figure 1A (left), each cancer type harbors mutations in the six SCF complex member genes that typically range from ~2% in ovarian cancer to ~38% in uterine cancer. Additionally, cursory analyses revealed that the core SCF complex member genes typically harbor fewer than 20 mutations within a given cancer type (Figure 1A; right), with specific genes, namely *SKP1* and *RBX1* having fewer than 10 mutations in each cancer type. Conversely, *CUL1* exhibits a higher mutational load in some cancers, as evidenced by the 23 and 35 mutations observed in stomach and uterine cancers, respectively. Similarly, the three F-box protein genes typically harbor few mutations, (1–20 total mutations) within most cancer types (Figure 1A; right); however, *FBXW7* is the most frequently mutated gene with 107 and 117 cases in colorectal and uterine cancers, respectively. Collectively, these data reveal that SCF complex member genes are mutated in a diverse array of cancer types with *FBXW7* being the most frequently mutated gene in many cases.

Next, to determine whether SCF complex member genes predominantly exhibit specific mutational subtypes (i.e., frameshift deletion/insertion, fusion, in-frame deletion/insertion, missense, nonsense or splice site/regions), TCGA sequencing data [35,36,37] were scrutinized for each of the six genes. The mutational frequencies and the predicted functional impacts of the single nucleotide polymorphisms (SNPs) (“benign”, “possibly damaging” and “probably damaging”) were determined using Sorting Intolerant From Tolerant (SIFT) and Polymorphism Phenotyping V2 (PolyPhen-2) (detailed in Materials and Methods). Unfortunately, PolyPhen-2 and SIFT score estimates are derived from protein alignments utilizing different protein databases, which sometimes results in inconsistent predictions between approaches. Accordingly, only PolyPhen-2 data were employed for mutation classification analyses, where missense mutations with “not available” (N/A) prediction scores were excluded from all downstream analyses. In general, the overall (Total) distributions of the various mutation categories (Figure 1B) are predominantly enriched for “probably damaging” mutations (42.4%), followed by “benign” (20.6%) and nonsense (14.8%) mutations, while individual genes are more variable. For example, *SKP1* predominantly harbors missense “benign” mutations (47.8%), while other mutations (i.e., missense “probably” damaging; 8.7%) are less common. *CUL1* exhibits numerous missense mutations (36.6% “probably damaging”; 32.41% “benign”), while a majority of *SKP2* mutations are missense “benign” mutations (47.4%). In contrast, *FBXW7* SNPs are primarily missense “probably damaging” mutations (54.5%), whereas *FBXO5* alterations are largely comprised of missense “benign” mutations (40%). In summary, while there is variation in the frequency of each mutational class between the six genes, there is an overall bias towards “probably damaging” missense mutations that supports the possibility that mutations within SCF complex member genes may have adverse implications for complex function and disease pathogenesis.

### 2.2. The Distributions of Encoded SCF Complex Alterations Are Consistent with a Tumor Suppressor Mutational Load

**Figure 2:**
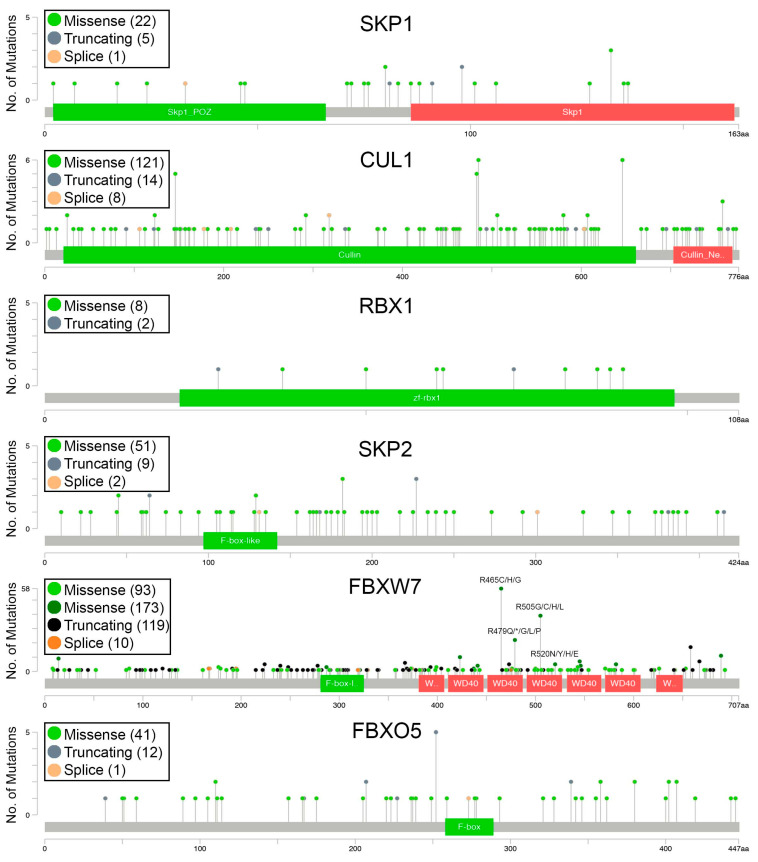
Distribution of the encoded alterations of six SCF complex members. Schematics depicting the amino acid position (*x*-axis) and frequency (*y*-axis) of encoded alterations from 10 cancer types, with the total number of each mutational subtype presented within brackets in the associated bounding box. Key protein motifs of SCF complex members are denoted in green and red. Note that light colors correspond to mutations of unknown significance and dark colors correspond to putative driver mutations (* indicates a premature stop codon).

To gain a greater insight into the overall distribution of encoded missense, truncating and splice site alterations within the SCF complex members, the frequency and location (i.e., amino acid position) of encoded alterations were assessed in 10 cancer types (Figure 2; Table S2). In general, all SCF complex members exhibit alterations that span the length of the encoded protein, an overall distribution that is consistent with a tumor suppressor mutational load [38]. Interestingly, however, several alteration hot-spots were observed in FBXW7, a feature that is more commonly associated with oncogenes [38] (Figure 2). For example, encoded alterations involving R465C/H/G occur in 58 cancer patient samples (bladder, breast, colorectal, head and neck, stomach and uterine). Similarly, the encoded alterations R479Q/G/L/P occur in 22 patient samples (bladder, breast, colorectal, head and neck, pancreatic, stomach and uterine cancers). R505G/C/H/L substitutions account for 39 of the encoded alterations, in colorectal, head and neck, ovarian, stomach and uterine cancers, whereas D520N/Y/H/E occurs in 22 bladder, colorectal, head and neck and ovarian patient cases. Notably, the alteration hotspots detailed above reside within the WD-40 domain of FBXW7, which in some cases may adversely impact protein structure (e.g., D520; discussed further below). Taken together, these data suggest that FBXW7 (and perhaps other SCF complex members) may exhibit tumor suppressor and/or oncogene-like functions in a context/tissue-dependent manner.

### 2.3. Encoded Alterations in SCF Complex Members May Adversely Impact Protein Structure

**Figure 3:**
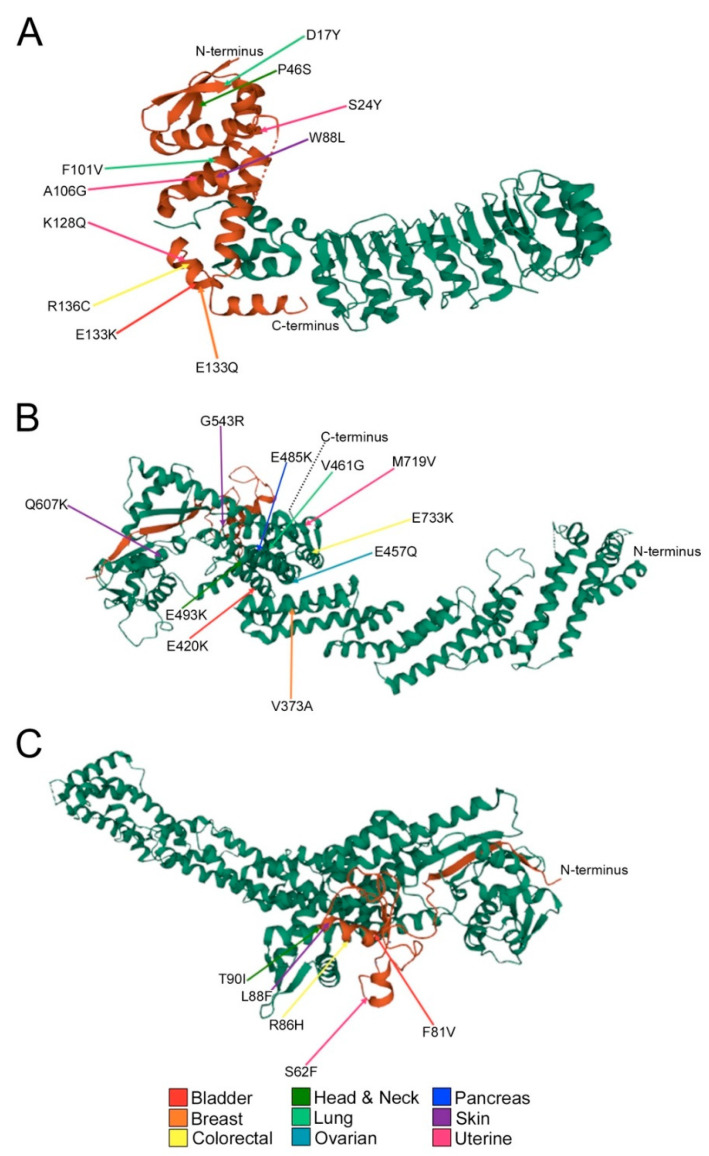
Missense mutations underlie potentially damaging alterations in core SCF complex members. (**A**) Partial crystal structure (missing amino acids 1-14; ribbon model) of the SKP1 (brown)-SKP2 (green) complex shows predicted detrimental alterations. Indicated amino acid substitutions are deemed deleterious, possibly damaging or probably damaging by SIFT and PolyPhen-2 databases (Table S2) and are predicted to impact protein–protein interactions between SKP1 and CUL1 or SKP1 and SKP2. Amino acids are denoted by their single letter code, numbers indicate amino acid position in the SKP1 protein. Colored arrows identify cancer type in which the underlying mutation was identified (see key). (**B**) Partial crystal structure (missing amino acids 1-16; ribbon model) of the CUL1 (green)-RBX1 (brown) complex presents predicted detrimental alterations. Indicated alterations are deemed deleterious, possibly/probably damaging by SIFT and PolyPhen-2 and are predicted to impact protein–protein interactions between CUL1-RBX1 or CUL1-SKP1. (**C**) Partial crystal structure (missing amino acids 1-18; ribbon model) of the CUL1 (green)-RBX1 (brown) complex presents predicted detrimental alterations. Indicated alterations are deemed deleterious, possibly/probably deleterious by SIFT and PolyPhen-2 and are predicted to impact protein–protein interactions between RBX1-CUL1 or RBX1-E2 conjugase.

**Figure 4:**
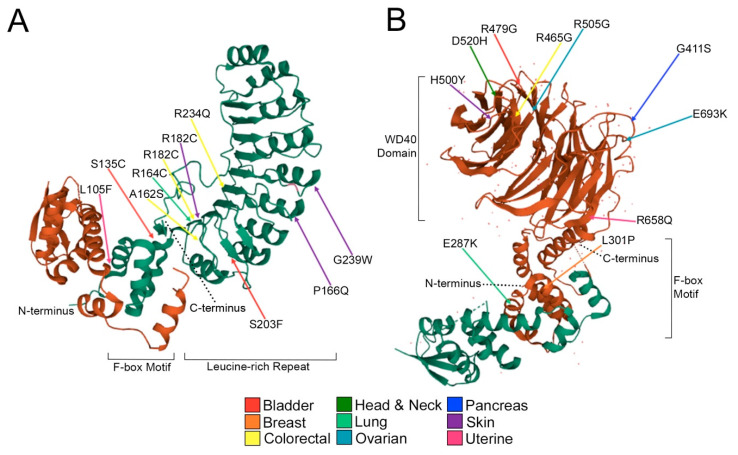
Missense mutations encode potentially detrimental amino acid substitutions in SKP2 and FBXW7. (**A**) Partial crystal structure (missing amino acids 1-88; ribbon model) of the SKP1 (brown)-SKP2 (green) complex presenting predicted damaging alterations. Indicated amino acid substitutions are deemed deleterious, possibly/probably damaging by SIFT and PolyPhen-2 (Table S2) and are predicted to impact protein–protein interactions between SKP1 and SKP2 or SKP2 and its target proteins. Amino acids are denoted by their single letter code, numbers indicate amino acid position in the SKP2 protein. Colored arrows identify cancer type in which the underlying mutation was identified (see key). (**B**) Partial crystal structure (missing amino acids 1-262; ribbon model) of the SKP1 (green)-FBXW7 (brown) complex presents predicted damaging alterations. Indicated alterations are deemed deleterious, possibly/probably damaging by SIFT and PolyPhen-2 and are predicted to impact protein–protein interactions between SKP1 and FBXW7 or FBXW7 and its substrate proteins.

Having described the frequency and location of encoded alterations in the preceding sections, we next sought to explore the potential impact these changes have on the structure and function by analyzing crystallographic data extracted from The Protein Data Bank (PDB) [31,39]. Mutations deemed “deleterious” or “possibly/probably damaging” by SIFT and Polyphen-2 (Table S2), respectively, are presented in Figure 3 and Figure 4. SIFT deems an alteration “deleterious” based on conserved amino acid positions, where alterations at strongly conserved positions are expected to be intolerant to most substitutions [40]. By contrast, PolyPhen-2 predictions are based on the stability and function of human proteins using functional annotation of SNPs, maps coding SNPs to gene transcripts, extracts protein sequence annotations and structural attributes [41]. Importantly, these specific amino acid substitutions could critically impact SCF complex formation and function by disrupting secondary structure, tertiary structure and protein–protein interactions.

Recall that SKP1 is an invariable member of the SCF complex that recruits the variable F-box proteins and their protein ligands [31]. Although SKP1 alterations occur most frequently in uterine and lung cancers, most substitutions only occur in a single cancer type, except for E133K/Q (bladder, breast; Figure 3A). Notably, ~50% of the encoded alterations deemed “deleterious” and “probably/possibly damaging” occur within the F-box protein recognition domain and affect net charge or alpha helicity including E133K/Q (bladder; breast), K128Q (uterine), R126C (colorectal) and A106G (uterine). CUL1 is the scaffolding member of the SCF complex [29] and alterations that potentially affect its ability to bind with either SKP1 or RBX1 could critically impact SCF complex formation and function. As with SKP1, most CUL1 alterations only occur within a given cancer type with the exceptions of E485K (bladder; head and neck; pancreatic; uterine) and Q607H/K (bladder; skin) (Figure 3B). Strikingly, ~50% of alterations involve glutamate residues that are frequently converted to lysine residues, resulting in a change in net charge and include E420K (bladder), E485K (uterine), E493K (head and neck), E733K (colorectal) and E457Q (lung). Only five RBX1 alterations were identified as “deleterious” or “possibly/probably damaging” across all patient samples including S62F (uterine), F81V (bladder), R86H (colorectal), L88F (skin) and T90I (head and neck) (Figure 3C). These alterations occur within the RBX1 zinc finger binding domain, which may impact interactions between RBX1 and the E2 enzyme or CUL1. Finally, FBXO5 is an F-box protein whose substrates remain largely unknown and has many “deleterious” and “possibly/probably damaging” alterations identified within colorectal and uterine cancers. Approximately half of the substitutions in colorectal cancer convert a leucine residue to a larger residue such as L205F, L220R and L259F. Conversely, there is a wide range of FBXO5 alterations in uterine cancer that includes a serine to glycine conversion within the F-box motif (S278G), which could potentially impact protein–protein interactions with SKP1 and the rest of the SCF complex. Unfortunately, there is no available crystal structure of SKP1-FBXO5 to date, therefore it is difficult to speculate how these alterations may impact either substrate interactions or SCF complex formation and function.

SKP2 is an F-box protein that has been primarily classified as an oncoprotein and targets the products of proto-oncogenes such as Cyclin E1 and c-MYC for proteolytic degradation [7,26,42,43]. *SKP2* mutations occur most frequently in bladder, colorectal, skin and uterine cancers and result in key amino acid substitutions, including L105F (uterine) and S135C (bladder; Figure 4A), that are located within the F-box motif, which is critical for interactions between SKP2 and the SCF complex [44]. Another important SKP2 protein domain is the leucine-rich repeat, consisting of a hydrophilic cap and a hydrophobic concave surface that are essential for ligand interaction [45]. Therefore, non-conservative alterations within this region may impact the ability of SKP2 to recognize and ubiquitinate protein targets. Substitutions in the leucine-rich repeat include the elimination of arginine residues (positively charged aliphatic chain) and consist of R164C (lung), R182C (colorectal; skin; uterine) and R234Q (colorectal). Other alterations in this domain include S203F (bladder), P166Q and G239W (uterine). Importantly, amino acid substitutions within SKP2 have the potential to impede normal function which can result in the aberrant accumulation of its substrate proteins.

FBXW7 is an F-box protein with a WD-40 protein domain that is critical for substrate specificity and whose target proteins includes cell cycle regulators and oncoproteins such as Cyclin E1 and c-MYC [26,46,47]. Unlike *SKP2*, there are “deleterious” and “possibly/probably damaging” substitutions in all 10 cancer types assessed, with the highest incidences occurring in breast, head and neck and uterine cancers. Substitutions within the FBXW7 F-box motif include E287V/K (colorectal; lung; stomach), which induces a change in net charge from negative to positive and L301P (breast), which is predicted to disrupt alpha helicity (Figure 4B). As with SKP2, there is a trend for arginine substitutions to occur across a diverse array of cancer types (bladder; colorectal; head and neck; stomach; uterine). These positively charged hydrophilic residues often protrude from the protein surface to participate in hydrogen bonding Van der Waals interactions in addition to aiding in salt bridge formation [48,49]. Therefore, arginine substitutions could critically impact the ability of the SCF complex to recognize substrates and regulate the abundance of target proteins. Notably, three of the four arginine substitutions are conversions to glycine residues, all occurring in close spatial proximity of one another (40 residues), at the exterior of the WD-40 ligand binding domain, which effectively eliminates a net positive charge by replacing it with a small hydrophobic residue. Additional substitutions in the WD-40 domain include changes in net charge (E693K; ovarian), gain of a hydrogen bonding group (G411S; pancreatic), or both (D520N/Y/H/E; bladder; colorectal; head and neck; ovarian). Collectively, these encoded amino acid substitutions are predicted to adversely impact the F-box proteins from interacting with the SCF complex and/or their protein substrates, which is expected to promote the aberrant accumulation of substrates and contribute to early disease development in certain instances.

### 2.4. SCF Complex Members Exhibit Frequent Copy Number Alterations in Cancer

**Figure 5:**
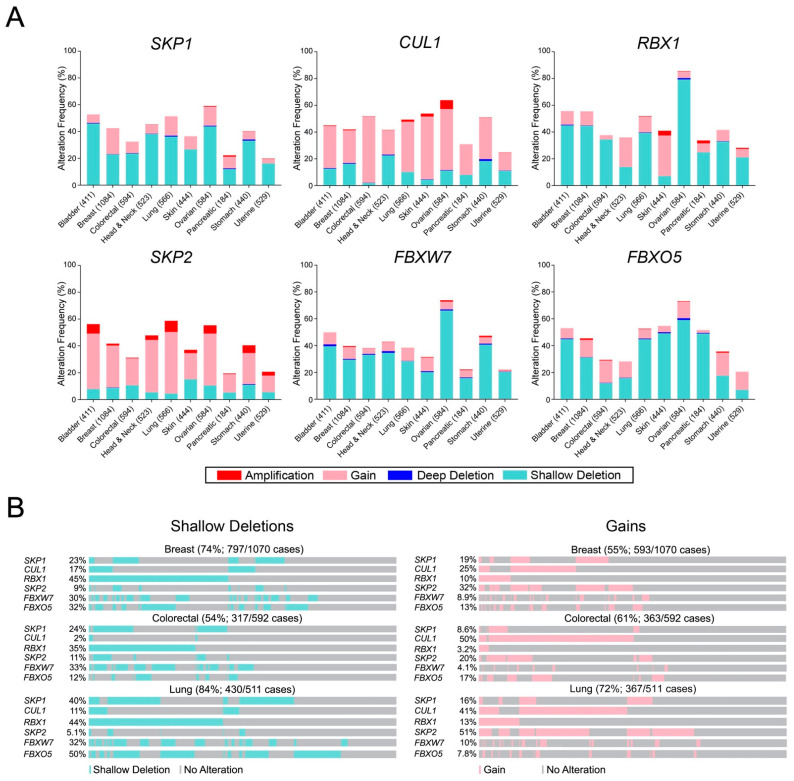
The frequency of gene copy number alterations of SCF complex members in 10 cancer types. (**A**) Bar graphs depicting the frequency of CNAs (deep deletions; shallow deletions; gains; amplifications) for six SCF complex member genes in 10 cancer types (Table S3). Cancer types are listed along the *x*-axis with the number of cases of each indicated in brackets. (**B**) OncoPrint data for breast, colorectal and lung cancer depicting the individual and cumulative frequencies for only shallow deletions (left) and gains (right) of six SCF complex member genes. Vertical alignments within a given cancer type identify samples from the same patient; patient-specific comparisons cannot be made between categories (i.e., shallow deletions versus gains).

While the preceding sections focused on specific mutations and their encoded amino acid substitutions within the six SCF complex members, we sought to investigate the prevalence of gene CNAs, which are expected to impact their overall expression levels. Like the previous sections, TCGA data [35,36,37] from 10 cancer types were scrutinized for CNAs including deep deletions, shallow deletions, gains and amplifications as described within Materials and Methods, with the full list of CNAs provided in Table S3. In general, CNAs occur frequently in all 10 cancer types and collectively range from ~19% (pancreatic) to ~85% (ovarian) (Figure 5A). More specifically, *SKP1* and *RBX1* predominantly exhibit shallow deletions that range from ~12% (pancreatic) to ~46% (bladder) and ~7% (skin) to ~79% (ovarian), respectively. In contrast, copy number gains occur most frequently with *CUL1* and range from ~13% (uterine) to ~50% (colorectal), whereas amplifications are rare, ranging from ~1.5% (lung) to ~7% (ovarian). *SKP2* also exhibits amplifications ranging from ~0.5% (colorectal) to ~8% (lung) and a prevalence of gains ranging from ~12% (uterine) to ~46% (lung). Conversely, *FBXW7* and *FBXO5* typically harbor shallow deletions ranging from ~15% (pancreatic) to ~66% (ovarian) and ~7% (uterine) to ~59% (ovarian), respectively. Collectively, these data show that *CUL1* and *SKP2* predominantly exhibit copy number gains (gains and amplifications), whereas *SKP1*, *RBX1*, *FBXW7* and *FBXO5* tend to exhibit more copy number losses (shallow and deep deletions). Importantly, these data lend further support to the possibility that aberrant expression of SCF complex member genes adversely impacts complex function and may contribute to the development and/or progression of many cancer types.

**Figure 6:**
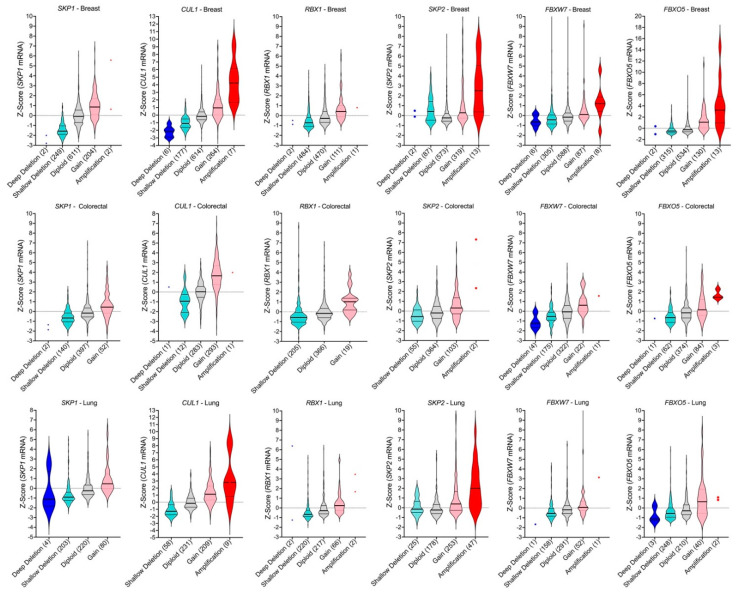
SCF complex copy number alterations correspond with changes in mRNA expression levels. Violin plots of mRNA expression data [35,36,37] from the three commonly diagnosed cancers (i.e., breast [top row], colorectal [middle] and lung [bottom]). *SKP1*, *CUL1*, *RBX1*, *SKP2*, *FBXW7*, and *FBXO5* CNAs (deep deletions; shallow deletions; gains, amplifications) and diploid cases are presented along the *x*-axis with total case numbers indicated within brackets. Note that categories with ≤ 2 cases are identified by dots and that in general, deep deletions, and amplifications are rare. Note that some of the *y*-axes are differentially scaled to better present the data.

It should also be noted that there is a compounding effect when the individual frequencies of shallow deletions and gains are concurrently assessed for the six genes. That is, the combined frequencies are >50% for losses and gains in breast, colorectal and lung cancers, the three most prevalent cancer types (Figure 5B). More specifically, the combined frequencies of shallow deletions are 54% (colorectal), 74% (breast) and 84% (lung), while combined frequencies of gains are 55% (breast), 61% (colorectal) and 72% (lung), which suggests that aberrant SCF complex expression (i.e., decreases or increases) may be a significant, yet underappreciated contributor to disease pathology in these cancer types. In support of this possibility, mRNA expression levels are significantly altered (reduced or increased) in a manner consistent with the specific CNA (i.e., loss or gain). For example, reduced mRNA expression is typically associated with copy number losses (deep and shallow deletions) for *SKP2*, *FBXW7* and *FBXO5* relative to the diploid state, while increased expression typically occurs with copy number gains (gains and amplifications) (Figure 6). Consistent with these findings, CNAs involving *SKP1*, *CUL1* and *RBX1* also correspond with overall changes in mRNA expression levels. Collectively, these data show that CNAs and corresponding changes in mRNA expression occur frequently for SCF complex member genes, highlighting how CNAs likely adversely affect SCF complex expression and/or function.

### 2.5. SCF Complex Members Are Differentially Methylated in Cancer

**Figure 7:**
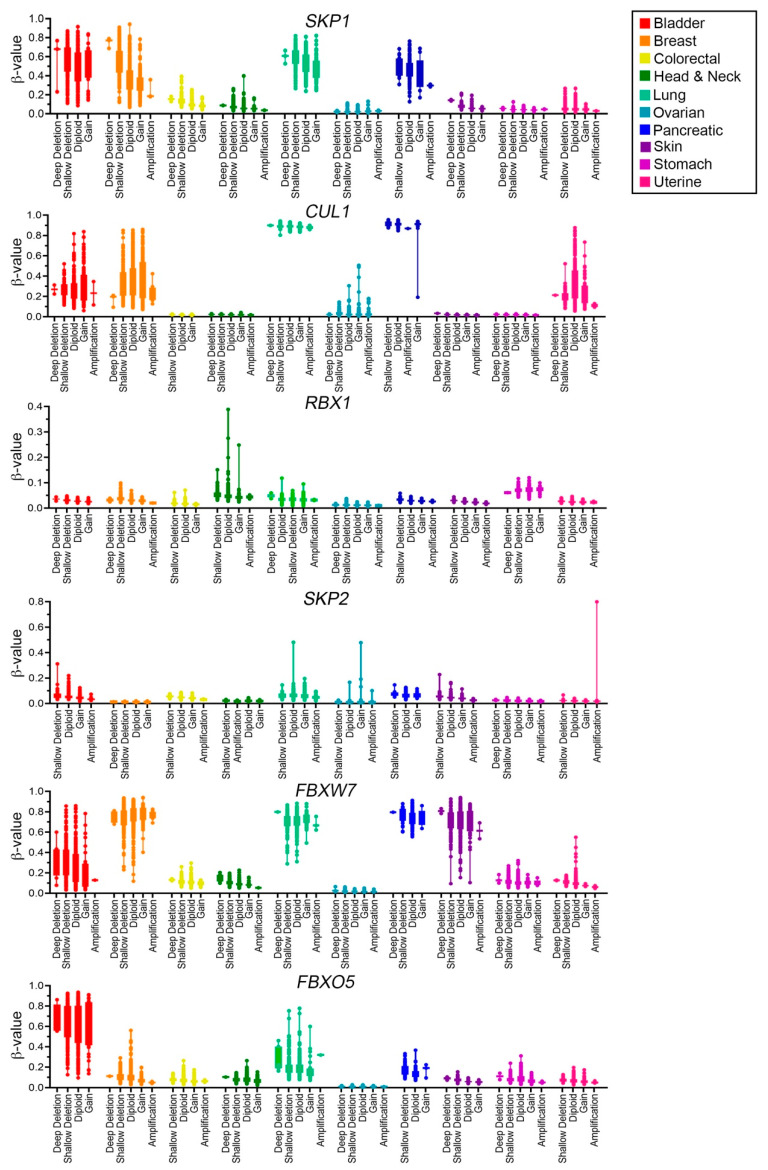
The SCF complex is differentially methylated in human cancers. Box-and-whisker plots presenting the methylation status of cancer samples derived from TCGA Firehose Legacy data (https://gdac.broadinstitute.org/, accessed on 18 November 2021) (see Tables S4–S9). Associated β-values of SCF complex members are presented along the *y*-axis. Note that the *y*-axis scale for *RBX1* and *SKP2* (0.4 and 0.8, respectively) differ relative to the remaining genes (1.0) for better data visualization. Methylation data are grouped according to gene CNAs (deep deletion; shallow deletion; gain; amplification) or diploid status (*x*-axis).

To assess the methylation status of SCF complex member genes, β-values and corresponding CNA data were exported from the TCGA Firehose Legacy dataset (https://gdac.broadinstitute.org/, accessed on 20 October 2021) using cBioPortal [35,36] (Figure 7; Tables S4–S9). Samples were characterized as hypomethylated (β < 0.2), partially methylated (0.2 ≤ β ≤ 0.7), or hypermethylated (β > 0.7) as described within Materials and Methods. Overall, SCF complex members exhibit diverse methylation profiles across the 10 cancer types. As expected, the methylation status of *SKP1* varies by cancer type, with *SKP1* being hypomethylated (i.e., expressed) in the majority of colorectal, head and neck, ovarian, stomach and uterine cases, whereas it tends to be partially or hypermethylated (i.e., repressed) in bladder, breast, lung and pancreatic cancers. Note that for all cancer types investigated, samples exhibiting *SKP1* copy number losses also tend to correspond with hypermethylated states (i.e., greater β-values) relative to diploid samples or those harboring copy number gains (Figure 7, Table S4). Overall, the methylation status of *CUL1* is very similar to that of *SKP1*, as tumor samples with copy number losses also tend to be hypermethylated; however, *CUL1* is generally partially methylated or hypermethylated in all uterine cancer cases (Figure 7, Table S5). Interestingly, *RBX1* (Figure 7, Table S6) and *SKP2* (Figure 7, Table S7) tend to be hypomethylated across all cancer types, irrespective of copy number status, whereas *FBXW7* is differentially methylated across cancer types (Figure 7, Table S8). Briefly, *FBXW7* is hypomethylated in colorectal, head and neck, ovarian, stomach and uterine patient samples and partially or hypermethylated in bladder, breast, lung, pancreatic and skin cancers. Finally, *FBXO5* is partially methylated in bladder and lung cancers, but is predominantly hypomethylated in all remaining cancer types (Figure 7, Table S9). As methylation is a negative regulator of gene expression [50], increases in methylation status may phenotypically mimic copy number losses and/or result in loss of heterozygosity, whereas aberrant decreases in methylation status are expected to correspond with increases in gene expression, which may phenocopy, to a limited extent, copy number gains. Thus, aberrant increases or decreases in methylation status may adversely impact SCF complex members harboring tumor suppressor [9,10,51,52] or oncogene-like functions [53,54], respectively. Collectively, these data show that SCF complex member genes are differentially methylated across a variety of cancer types and supports the possibility that changes in methylation status may induce aberrant SCF complex function and contribute to cancer pathogenesis.

## 3. Discussion

In this study, we examined the genetic and epigenetic changes associated with six prototypic SCF complex member genes across 10 common cancer types. First, we determined that each member is somatically mutated across multiple cancer types, with *FBXW7* exhibiting the most frequent number of mutations. Additionally, we determined that the mutations are typically distributed across the entirety of their respective coding regions, which is consistent with the mutational distribution of a tumor suppressor gene [38]; however, *FBXW7* also harbored focal hot-spots that are consistent with the mutational profile of an oncogene [38]. Interestingly, in silico analyses determined that many of the encoded amino acid substitutions are predicted to adversely impact protein structure and/or function of the SCF complex, supporting a potential role in cancer pathogenesis. Next, CNA analyses revealed that each gene is frequently altered in 10 common cancer types, and that *SKP1*, *RBX1*, *FBXW7* and *FBXO5* tend to exhibit more losses, while *CUL1* and *SKP2* exhibit more gains. It is also important to note that the specific categories of CNAs corresponded with similar changes in gene expression at the mRNA levels. That is, cancers with deep or shallow deletions exhibited reduced mRNA expression levels relative to diploid cases, while those harboring gains or amplifications exhibited increases. Finally, examination of their DNA methylation profiles revealed a large range of patterns for each gene. In general, samples harboring copy number losses (particularly shallow deletions) were frequently hypermethylated relative to the diploid cases or those with copy number gains (gains or amplifications). In summary, these findings determined that the six SCF complex member genes exhibit frequent mutations, CNAs and/or aberrant methylation profiles that collectively are predicted to adversely impact complex member expression and/or function that is consistent with an etiological role in cancer development and progression.

When assessing the amino acid substitutions in conjunction with available crystal structure data, our results highlight the potential functional impact that encoded alterations may have on the SCF complex and identifies a possible mechanism by which aberrant SCF complex structure and function may contribute to cancer pathogenesis. Importantly, additional functional studies are highly warranted as not every mutation (and encoded alteration) will have pathogenic implications in cancer; however, it is well established that specific amino acid substitutions can adversely impact protein structure and function, and thus have the potential to contribute to disease pathogenesis. For example, the mutations we identified that alter key amino acid characteristics, including net charge, hydrogen bonding ability and steric interactions (i.e., protein–protein interactions) within the leucine-rich repeat domain of SKP2 or the WD-40 domain of FBXW7 can decrease the affinity and specificity of these binding sites for their protein substrates, including the oncoproteins Cyclin E1 and c-MYC [26,47,55]. SKP2 also targets P27 (*CDKN1B*) for proteolytic degradation, a cell cycle regulating protein that inhibits Cyclin Dependent Kinase activity [56]. Importantly, aberrant P27 accumulation is associated with chromosome instability (CIN; ongoing changes in chromosome numbers) and mitotic defects [2,53,57], whereas aberrant Cyclin E1 and c-MYC turnover lead to cell cycle and apoptotic defects that promote cancer development and progression [9,10,12,58,59,60,61]. In fact, increased *c-Myc* expression in mouse embryonic fibroblasts increases growth rate, enhances clonogenic growth, decreases in contact inhibition and spontaneously forms tumors in mice within 10 days [62], providing strong evidence that deregulated *c-MYC* expression exhibits a significant role in tumorigenesis. Importantly, while the c-MYC protein is overexpressed in ∼70% of human cancers, typically only ~20% of tumors harbor *c-MYC* gene amplifications or translocations and thus it remains possible that aberrant c-MYC turnover/degradation may account for this discrepancy [63]. Thus, these findings highlight the importance of c-MYC regulation at the protein level and the possible malignant consequences of abnormal SCF complex function. Moreover, the detrimental impact encoded alterations have on protein structure and function is perhaps best exemplified by Wang and colleagues [55], who determined that D331 within the leucine-rich repeat domain of SKP2 is essential for its interaction with CKS1 (Cyclin-dependent Kinases regulatory protein 1) and the subsequent ubiquitination of P27. More specifically, they noted that a D331A substitution converted the normally negative surface potential to a positive potential, which ablated the SKP2-CKS1 interaction, while a conserved substitution, D331E, maintained the interaction. Similarly, amino acid substitutions involving R417 and R457 within the WD40 domain of FBXW7 abolish Cyclin E binding, while R495 substitutions reduce binding affinity [47,64]. While none of these alterations were detected within the current study, their results highlight the need and value of downstream genetic and functional studies aimed at assessing the plethora of individual substitutions to accurately determine their impacts on protein–protein interactions and overall SCF complex function. This is particularly important as the SCF complex regulates numerous biological processes required for genome stability, including cell cycle progression, centrosome biology and DNA repair [9,10,12,19,20,65]. Thus, it is conceivable that specific amino acid substitutions occurring in key protein motifs may adversely impact SCF complex function and underlie the accumulation of protein substrates, especially (proto-) oncogenes whose increase in abundance promotes genome instability and contributes to cancer pathogenesis [9,10,12,19,20]. For example, *CCNE1* (Cyclin E1) is an established oncogene [58,66,67] that is genomically amplified in many cancer types [35,36,37] and whose overexpression induces CIN, which is associated with cell cycle misregulation, genome instability, cellular transformation and tumor formation in mice [59]. More specifically, Karst and colleagues [67] determined that increased abundance of Cyclin E1 leads to inappropriate cell growth including accelerated growth, loss of contact-inhibition, clonogenic growth and anchorage independent growth. Moreover, we recently demonstrated that reduced expression of *SKP1*, *CUL1* and *RBX1* prevents Cyclin E1 degradation leading to an increase in abundance that induces CIN and promotes cellular transformation in colorectal and ovarian cancer contexts [9,10,12]. As the three core SCF complex members interact in an epistatic manner, it is reasonable to assume that specific alterations to any of these proteins will adversely impact complex formation and lead to abnormal increases in Cyclin E1 abundance. Additionally, it was also shown through the use of genetic rescue experiments that the aberrant increases in Cyclin E1 observed following *FBXW7* inactivation in colorectal cancer cells were an underlying mechanism driving increases in CIN [68]. Importantly, SKP2 and FBXW7 regulate Cyclin E1 turnover [2,18,25,46] and thus specific *SKP2* and *FBXW7* mutations, CNAs or altered methylation (i.e., hypermethylation) status are predicted to adversely impact Cyclin E1 abundance and contribute to disease pathogenesis. Thus, it is the collective lack of Cyclin E1 turnover arising from defects in the expression and/or function of SCF complex members that is expected to phenocopy genomic amplification of *CCNE1* and contribute to cancer pathogenesis. Given estimates that the SCF complex targets hundreds to thousands of proteins, it is possible that the aberrant regulation (i.e., turnover) of additional protein substrates, such as P27, RAD51, c-MYC may also contribute to cancer pathogenesis [3,4,5,7,43,69].

While TCGA data reveal that CNAs of the six SCF complex member genes are common in cancer and are either predominantly gained or lost, it is important to highlight the phenotypic consequences that these CNAs may have in cancer irrespective of their traditionally established oncogenic or tumor suppressive roles. Previous genetic studies have shown that heterozygous knockout of *SKP1*, *CUL1*, *RBX1* and *FBXO7* induces CIN [9,10,11], an enabling hallmark of cancer [70] frequently associated with cellular transformation, drug resistance, metastasis and poor patient prognosis in many cancer types [71,72,73,74,75]. Indeed, CIN is a dynamic form of genome instability that drives ongoing changes in genetic and cell-to-cell heterogeneity that is proposed to contribute to cancer development by increasing the rate at which oncogenes, tumor suppressor genes and cell cycle regulators are gained, lost, or altered [76,77,78]. Importantly, the phenotypic consequences corresponding with cancer development (e.g., CIN) following aberrant expression of SCF complex members may vary and are likely to arise via the misregulation of protein substrates in a context-dependent manner. For example, overexpression of SCF complex members may result in excess degradation of protein substrates with tumor suppressor functions, and has been associated with cancer cell stemness, tumor progression and worse patient survival [79,80,81,82,83,84,85,86]. Similarly, amplification and overexpression of *SKP2* promotes the increased degradation of P27 and disease progression [56] and is associated with worse survival outcomes and poor response to therapy in numerous cancer types [87,88,89], contributing to *SKP2* being traditionally classified as an oncogene. Conversely, other studies have shown that reduced *SKP2* expression leads to aberrant increases in P27 abundance that is suggested to prevent mitotic entry and result in increased nuclear areas [2], a CIN-associated phenotype [70,90,91,92] suggestive of large increases in DNA/chromosome content (i.e., polyploidy) [69]. In contrast, *FBXW7* has been primarily classified as a tumor suppressor gene [51,52] and loss of SCF^FBXW7^ activity corresponds with aneuploidy, micronucleus formation (extranuclear bodies found outside the primary nucleus and a hallmark of CIN), sister chromatid cohesion defects and chromosome segregation defects (i.e., CIN) [68,93]. However, our data support the possibility that *FBXW7* may also exhibit oncogene-like roles as gains, amplifications and mutational hot-spots were observed [38]. Indeed, Galindo-Moreno and colleagues [94] determined that *FBXW7* amplification in tumors harboring wild-type *TP53* expression reduced breast cancer patient survival but did not impact survival of patients with skin or bladder cancers. Accordingly, these collective data highlight that losses or gains of *FBXW7* (and perhaps other SCF complex member genes), may adversely contribute to cancer pathogenesis in a context-dependent manner [95]. With respect to *FBXO5*, Marzio et al. [3] recently determined that reduced *FBXO5* expression corresponds with a DNA re-replication phenotype (i.e., endoreduplication) in a breast cancer model and that loss of its F-box dependent function leads to increases in RAD51 abundance (a homologous recombination repair protein). In contrast, Vaidyanathan and co-workers [96] determined that *FBXO5* overexpression corresponds with increases in mitotic defects (e.g., lagging and incorrect segregation of chromosomes) and aneuploidy that promotes CIN in transgenic mouse models. Additionally, it is important to note that while each F-box protein targets a distinct array of protein substrates, there is some functional redundancy between F-box proteins, in that multiple F-box proteins can share specific substrates. For example, while SKP2 and FBXW7 exhibit distinct substrate profiles, both target Cyclin E1 and c-MYC for proteolytic degradation [2,7,26,43]. Thus, this inherent functional redundancy may serve as a compensatory mechanism to limit the functional outcomes associated with the aberrant expression and/or function of a given F-box protein. Therefore, with the knowledge that 69 distinct SCF complexes are predicted to target hundreds to thousands of protein substrates, aberrant expression of the core SCF complex members are expected to evoke greater phenotypic consequences than alterations involving the individual F-box proteins. In this regard, future functional studies are required to determine the impacts aberrant expression and function of each individual SCF complex member have on protein substrates, genome instability and disease pathogenesis are now highly warranted.

In summary, this study revealed that the six SCF complex member genes predominantly exhibit missense damaging mutations, a subset of which likely impact SCF complex function by disrupting protein–protein interactions between SCF complex members and their protein substrates. Further, the distribution of the encoded amino acid substitutions and CNA data support the possibility that SCF complex members may exhibit both tumor suppressive or oncogenic roles that will likely be disease and context dependent. Finally, we determined that SCF complex members tend to be differentially methylated across multiple cancer types, which may phenotypically mimic certain CNAs (e.g., shallow deletions) and contribute to cancer pathogenesis but in an epigenetic manner. Ultimately, these alterations may underlie the aberrant accumulation of protein substrates that are implicated in CIN [2,9,10,12,59] and cancer pathogenesis [3,7,26,67,97,98]. Unfortunately however, the complete spectrum of target specificities of all 69 F-box proteins and their implications for disease development remain largely unknown or poorly characterized. Accordingly, functional studies are now required to determine the specific outcomes driven by aberrant expression and/or function of each SCF complex member gene and its impact on substrate accumulation to better understand the molecular determinants giving rise to cancer development, which will be critical to develop innovative precision medicine strategies to better combat the disease. For instance, as aberrant SCF complex function is associated with CIN [9,10,12], developing biomarkers to detect abnormal SCF complex expression could aid early diagnosis in cancers that exhibit high levels of CIN, including colorectal [99] and ovarian cancers [100,101], where early disease detection will be critical to ultimately improve patient outcomes [102,103]. Thus, future studies need to be designed that compile and analyze patient sample datasets for mutations, CNAs and methylation changes with matched normal samples and patient outcomes to gain a comprehensive understanding of how genetic alterations are associated with disease and their impact on patient outcomes. In addition, studies aimed at investigating the pathogenic implications of SCF complex alterations are needed to gain a greater understanding of the etiological events contributing to oncogenesis.

## 4. Materials and Methods

### 4.1. Genomic Datasets and Data Collection

Two publicly available TCGA patient-based datasets were utilized for this study, including TCGA Pan-Cancer Atlas [37] (mutation data and gene CNAs) and TCGA Firehose Legacy (methylation data; https://gdac.broadinstitute.org/, accessed on 20 October 2021). Genetic analyses were performed in cBioPortal (https://www.cbioportal.org, accessed on 20 October 2021) [35,36] for SCF complex members *SKP1*, *CUL1, RBX1*, *SKP2*, *FBXW7* and *FBXO5*. Patient datasets included bladder urothelial carcinoma, breast invasive carcinoma, colorectal adenocarcinoma, head and neck squamous cell carcinoma, lung adenocarcinoma, ovarian serous cystadenocarcinoma, pancreatic adenocarcinoma, skin cutaneous melanoma, stomach adenocarcinoma and uterine corpus endometrial carcinoma patient samples. All TCGA datasets were accessed no later than 10 November 2021.

### 4.2. Assessing the Frequency, Distribution and Predicted Functional Impact of Encoded SCF Complex Mutations

SCF complex member SNPs were evaluated to assess the frequency and the predicted functional impact of encoded alterations. Mutational frequencies were calculated as follows: total mutations within a given mutation class (n)/total number of mutations (N) for each gene × 100%. SNPs were assessed using SIFT (https://sift.bii.a-star.edu.sg/, accessed on 9 November 2021) [104] and PolyPhen-2 (http://genetics.bwh.harvard.edu/pph2/) [105]; Note that only single amino acid substitutions can be assessed using PolyPhen-2 and SIFT, where the remaining alterations (e.g., fusions, splice sites) are denoted with not applicable (N/A). For reference purposes, SIFT classifies SNPs as tolerated or deleterious [104], whereas PolyPhen-2 classifies SNPs as benign, possibly damaging or probably damaging [105]. Conceptually, SIFT assesses the conservation of an amino acid position (i.e., sequence identity/similarity) across species, where substitutions occurring at highly conserved positions that are deemed “deleterious” [40]. By contrast, PolyPhen-2 predicts the impact of substitutions on the stability and function of proteins using functional annotation of SNPs, maps coding SNPs to gene transcripts, extracts protein sequence annotations and structural attributes to create a score ranging from 0.0 (benign) to 1.0 (damaging) to ultimately assign it as “benign”, “possibly damaging” or “probably damaging”. A prediction of “possibly damaging” means that the substitution is predicted to be damaging, but with low confidence [41]. To display the spatial distribution and category of the encoded alterations (e.g., missense, truncating, splice or fusion), lollipop diagrams were extracted from cBioPortal [35,36,37] and alterations were classified as: (1) missense (unknown significance); (2) missense (putative driver); (3) truncating (unknown significance); (4) truncating (putative driver); (5) splice (unknown significance); or (6) or splice (putative diver). Figures presenting mutational data were generated with Prism v9 (GraphPad, San Diego, CA, USA) and assembled in Photoshop 2022 (Adobe, Toronto, ON, Canada).

### 4.3. Assessing the Predicted Structural Impact of Encoded SCF Complex Alterations

Crystal structures for SCF complex members were retrieved and visualized using the PDB (https://www.rcsb.org/, accessed on 9 November 2021) [39]: (1) SKP1 (PDB ID: 1FQV [31,39]); (2) CUL1 (PDB ID: ILDJ [39,106]); (3) RBX1 (PDB ID: ILDJ [39,106]); (4) SKP2 (PDB ID: 1FQV [31,39]); and (5) FBXW7 (PDB ID: 2OVP [39,107]). No crystal structure is available for FBXO5 (EMI1). All datasets were accessed no later than 10 November 2021. Figures were assembled in Photoshop.

### 4.4. Gene Copy Number Alteration and mRNA Expression Analyses

Gene CNA data were extracted from TCGA Pan-Cancer Atlas [37] with CNAs identified using the following Onco Query Language commands in cBioPortal [35,36]: (1) HOMDEL, deep deletion (i.e., loss of 2 alleles); (2) HETLOSS, shallow deletion (i.e., loss of 1 allele); (3) GAIN, small gain (i.e., gain of 1 allele); and (4) AMP, large amplification (i.e., gain of ≥ 2 alleles). OncoPrint and mRNA expression data were retrieved using cBioPortal (no later than 10 November 2021) to identify the individual and cumulative frequencies of CNAs (shallow deletions and gains) which were compared with their respective mRNA expression z-scores (z = expression in tumor sample-mean expression in reference sample[diploid])/standard deviation of expression in reference sample[diploid]) for breast, colorectal and lung cancers. Figures presenting CNA data were generated in Prism and assembled in Photoshop.

### 4.5. Assessing the Methylation Status of SCF Complex Members

The methylation status of each gene was assessed using beta (β) values and corresponding CNA data and extracted from TCGA Firehose Legacy (https://gdac.broadinstitute.org/) for each cancer type. Briefly, β-values were calculated as follows: β = M/M + U, where M > 0 and U > 0 represent the methylated and unmethylated signal intensities measured by the Illumina 27k (ovarian serous cystadenocarcinoma) or 450k BeadChip arrays (all other cancer types), respectively [108]. For each gene, samples were characterized as being hypomethylated (β < 0.2), partially methylated (0.2 ≤ β ≤ 0.7), or hypermethylated (β > 0.7). All data were accessed no later than 10 November 2021. Figures presenting methylation data were generated in Prism and assembled in Photoshop.

## Data Availability

Patient-related data from Figure 1, Figure 2, Figure 3, Figure 4, Figure 5, Figure 6 and Figure 7 are based on data available from TCGA Research Network (https://www.cancer.gov/tcga, accessed on 18 November 2021) and accessed online from 20 August to 10 November 2021.

## Supplementary Materials

The following are available online at https://www.mdpi.com/article/10.3390/ijms23010084/s1.

## References

[1] Sung H., Ferlay J., Siegel R.L., Laversanne M., Soerjomataram I., Jemal A., Bray F. (2021). Global cancer statistics 2020: GLOBOCAN estimates of incidence and mortality worldwide for 36 cancers in 185 countries. CA Cancer J. Clin..

[2] Nakayama K., Nagahama H., Minamishima Y.A., Matsumoto M., Nakamichi I., Kitagawa K., Shirane M., Tsunematsu R., Tsukiyama T., Ishida N. (2000). Targeted disruption of Skp2 results in accumulation of cyclin E and p27(Kip1), polyploidy and centrosome overduplication. EMBO J..

[3] Marzio A., Puccini J., Kwon Y., Maverakis N.K., Arbini A., Sung P., Bar-Sagi D., Pagano M. (2019). The F-Box Domain-Dependent Activity of EMI1 Regulates PARPi Sensitivity in Triple-Negative Breast Cancers. Mol. Cell.

[4] Duda D.M., Olszewski J.L., Tron A.E., Hammel M., Lambert L.J., Waddell M.B., Mittag T., DeCaprio J.A., Schulman B.A. (2012). Structure of a Glomulin-RBX1-CUL1 Complex: Inhibition of a RING E3 Ligase through Masking of Its E2-Binding Surface. Mol. Cell.

[5] Deshaies R.J. (1999). SCF and Cullin/RING H2-Based Ubiquitin Ligases. Annu. Rev. Cell Dev. Biol..

[6] Cepeda D., Ng H.F., Sharifi H.R., Mahmoudi S., Cerrato V.S., Fredlund E., Magnusson K., Nilsson H., Malyukova A., Rantala J. (2013). CDK-mediated activation of the SCFFBXO28 ubiquitin ligase promotes MYC-driven transcription and tumourigenesis and predicts poor survival in breast cancer. EMBO Mol. Med..

[7] Kim S.Y., Herbst A., Tworkowski K.A., Salghetti S.E., Tansey W.P. (2003). Skp2 regulates Myc protein stability and activity. Mol. Cell.

[8] Zhang Q., Spears E., Boone D.N., Li Z., Gregory M.A., Hanna S.R. (2013). Domain-specific c-Myc ubiquitylation controls c-Myc transcriptional and apoptotic activity. Proc. Natl. Acad. Sci. USA.

[9] Bungsy M., Palmer M.C.L., Jeusset L.M., Neudorf N.M., Lichtensztejn Z., Nachtigal M.W., McManus K.J. (2021). Reduced RBX1 expression induces chromosome instability and promotes cellular transformation in high-grade serous ovarian cancer precursor cells. Cancer Lett..

[10] Lepage C.C., Palmer M.C.L., Farrell A.C., Neudorf N.M., Lichtensztejn Z., Nachtigal M.W., McManus K.J. (2021). Reduced SKP1 and CUL1 expression underlies increases in Cyclin E1 and chromosome instability in cellular precursors of high-grade serous ovarian cancer. Br. J. Cancer.

[11] Palmer M.C.L., Neudorf N.M., Farrell A.C., Razi T., Lichtensztein Z., McManus K.J. (2021). The F-box protein, FBXO7 is required to maintain chromosome stability in humans. Hum. Mol. Genet..

[12] Thompson L.L., Baergen A.K., Lichtensztejn Z., McManus K.J. (2020). Reduced SKP1 expression induces chromosome instability through aberrant cyclin E1 protein turnover. Cancers.

[13] Hershko A., Ciechanover A. (1998). THE UBIQUITIN SYSTEM. Annu. Rev. Biochem..

[14] Loda M., Cukor B., Tam S.W., Lavin P., Fiorentino M., Draetta G.F., Jessup J.M., Pagano M. (1997). Increased proteasome-dependent degradation of the cyclin-dependent kinase inhibitor p27 in aggressive colorectal carcinomas. Nat. Med..

[15] Joazeiro C.A., Wing S.S., Huang H., Leverson J.D., Hunter T., Liu Y.C. (1999). The tyrosine kinase negative regulator c-Cbl as a RING-type, E2-dependent ubiquitin-protein ligase. Science.

[16] Scheffner M., Werness B.A., Huibregtse J.M., Levine A.J., Howley P.M. (1990). The E6 oncoprotein encoded by human papillomavirus types 16 and 18 promotes the degradation of p53. Cell.

[17] Waterman H., Levkowitz G., Alroy I., Yarden Y. (1999). The RING finger of c-Cbl mediates desensitization of the epidermal growth factor receptor. J. Biol. Chem..

[18] Nakayama K.I., Hatakeyama S., Nakayama K. (2001). Regulation of the cell cycle at the G1-S transition by proteolysis of cyclin E and p27Kip1. Biochem. Biophys. Res. Commun..

[19] Bassermann F., Eichner R., Pagano M. (2014). The ubiquitin proteasome system - Implications for cell cycle control and the targeted treatment of cancer. Biochim. Biophys. Acta.

[20] Thompson L.L., Rutherford K.A., Lepage C.C., McManus K.J. (2021). The SCF Complex Is Essential to Maintain Genome and Chromosome Stability. Int. J. Mol. Sci..

[21] Kleiger G., Mayor T. (2014). Perilous journey: A tour of the ubiquitin-proteasome system. Trends Cell Biol..

[22] Buetow L., Gabrielsen M., Huang D.T. (2018). Single-Turnover RING/U-Box E3-Mediated Lysine Discharge Assays. Methods Mol. Biol..

[23] Pickart C.M. (2001). Mechanisms Underlying Ubiquitination. Annu. Rev. Biochem..

[24] Thrower J.S. (2000). Recognition of the polyubiquitin proteolytic signal. EMBO J..

[25] Tetzlaff M.T., Yu W., Li M., Zhang P., Finegold M., Mahon K., Harper J.W., Schwartz R.J., Elledge S.J. (2004). Defective cardiovascular development and elevated cyclin E and Notch proteins in mice lacking the Fbw7 F-box protein. Proc. Natl. Acad. Sci. USA.

[26] Yada M., Hatakeyama S., Kamura T., Nishiyama M., Tsunematsu R., Imaki H., Ishida N., Okumura F., Nakayama K., Nakayama K.I. (2004). Phosphorylation-dependent degradation of c-Myc is mediated by the F-box protein Fbw7. EMBO J..

[27] Hussain M., Lu Y., Liu Y.Q., Su K., Zhang J., Liu J., Zhou G.B. (2016). Skp1: Implications in cancer and SCF-oriented anti-cancer drug discovery. Pharmacol. Res..

[28] Petroski M.D., Deshaies R.J. (2005). Function and regulation of cullin-RING ubiquitin ligases. Nat. Rev. Mol. Cell Biol..

[29] Dias D.C., Dolios G., Wang R., Pan Z.Q. (2002). CUL7: A DOC domain-containing cullin selectively binds Skp1·Fbx29 to form an SCF-like complex. Proc. Natl. Acad. Sci. USA.

[30] Cardozo T., Pagano M. (2004). The SCF ubiquitin ligase: Insights into a molecular machine. Nat. Rev. Mol. Cell Biol..

[31] Schulman B.A., Carrano A.C., Jeffrey P.D., Bowen Z., Kinnucan E.R., Finnin M.S., Elledge S.J., Harper J.W., Pagano M., Pavletich N.P. (2000). Insights into SCF ubiquitin ligases from the structure of the Skp1-Skp2 complex. Nature.

[32] Yoshida Y., Murakami A., Tanaka K. (2011). Skp1 stabilizes the conformation of F-box proteins. Biochem. Biophys. Res. Commun..

[33] Kamura T., Koepp D.M., Conrad M.N., Skowyra D., Moreland R.J., Iliopoulos O., Lane W.S., Kaelin W.G., Elledge S.J., Conaway R.C. (1999). Rbx1, a component of the VHL tumor suppressor complex and SCF ubiquitin ligase. Science.

[34] Silverman J.S., Skaar J.R., Pagano M. (2012). SCF ubiquitin ligases in the maintenance of genome stability. Trends Biochem. Sci..

[35] Cerami E., Gao J., Dogrusoz U., Gross B.E., Sumer S.O., Aksoy B.A., Jacobsen A., Byrne C.J., Heuer M.L., Larsson E. (2012). The cBio cancer genomics portal: An open platform for exploring multidimensional cancer genomics data. Cancer Discov..

[36] Gao J., Aksoy B.A., Dogrusoz U., Dresdner G., Gross B., Sumer S.O., Sun Y., Jacobsen A., Sinha R., Larsson E. (2013). Integrative analysis of complex cancer genomics and clinical profiles using the cBioPortal. Sci. Signal..

[37] Hoadley K.A., Yau C., Hinoue T., Wolf D.M., Lazar A.J., Drill E., Shen R., Taylor A.M., Cherniack A.D., Thorsson V. (2018). Cell-of-Origin Patterns Dominate the Molecular Classification of 10,000 Tumors from 33 Types of Cancer. Cell.

[38] Liu H., Xing Y., Yang S., Tian D. (2011). Remarkable difference of somatic mutation patterns between oncogenes and tumor suppressor genes. Oncol. Rep..

[39] Sehnal D., Bittrich S., Deshpande M., Svobodová R., Berka K., Bazgier V., Velankar S., Burley S.K., Koca J., Rose A.S. (2021). Mol* Viewer: Modern web app for 3D visualization and analysis of large biomolecular structures. Nucleic Acis Res..

[40] Ng P.C., Henikoff S. (2001). Predicting deleterious amino acid substitutions. Genome Res..

[41] Adzhubei I., Jordan D.M., Sunyaev S.R. (2013). Predicting functional effect of human missense mutations using PolyPhen-2. Curr. Protoc. Hum. Genet..

[42] Latres E., Chiarle R., Schulman B.A., Pavletich N.P., Pellicer A., Inghirami G., Pagano M. (2001). Role of the F-box protein Skp2 in lymphomagenesis. Proc. Natl. Acad. Sci. USA.

[43] Von Der Lehr N., Johansson S., Wu S., Bahram F., Castell A., Cetinkaya C., Hydbring P., Weidung I., Nakayama K., Nakayama K.I. (2003). The F-box protein Skp2 participates in c-Myc proteosomal degradation and acts as a cofactor for c-Myc-regulated transcription. Mol. Cell.

[44] Ng R.W.M., Arooz T., Yam C.H., Chan I.W.Y., Lau A.W.S., Poon R.Y.C. (1998). Characterization of the cullin and F-box protein partner Skp1. FEBS Lett..

[45] Xu C., Min J. (2011). Structure and function of WD40 domain proteins. Protein Cell.

[46] Strohmaier H., Spruck C.H., Kaiser P., Won K.A., Sangfelt O., Reed S.I. (2001). Human F-box protein hCdc4 targets cyclin E for proteolysis and is mutated in a breast cancer cell line. Nature.

[47] Koepp D.M., Schaefer L.K., Ye X., Keyomarsi K., Chu C., Harper J.W., Elledge S.J. (2001). Phosphorylation-dependent ubiquitination of cyctin E by the SCFFbw7 ubiquitin ligase. Science.

[48] Guccione E., Richard S. (2019). The regulation, functions and clinical relevance of arginine methylation. Nat. Rev. Mol. Cell Biol..

[49] Blanc R.S., Richard S. (2017). Arginine Methylation: The Coming of Age. Mol. Cell.

[50] Siegfried Z., Simon I. (2010). DNA methylation and gene expression. Wiley Interdiscip. Rev. Syst. Biol. Med..

[51] Akhoondi S., Sun D., von der Lehr N., Apostolidou S., Klotz K., Maljukova A., Cepeda D., Fiegl H., Dafou D., Marth C. (2007). FBXW7/hCDC4 is a general tumor suppressor in human cancer. Cancer Res..

[52] Yeh C.H., Bellon M., Nicot C. (2018). FBXW7: A critical tumor suppressor of human cancers. Mol. Cancer.

[53] Bretones G., Acosta J.C., Caraballo J.M., Ferrandiz N., Gomez-Casares M.T., Albajar M., Blanco R., Ruiz P., Hung W.C., Albero M.P. (2011). SKP2 oncogene is a direct MYC target gene and MYC down-regulates p27(KIP1) through SKP2 in human leukemia cells. J. Biol. Chem..

[54] Li C., Du L., Ren Y., Liu X., Jiao Q., Cui D., Wen M., Wang C., Wei G., Wang Y. (2019). SKP2 promotes breast cancer tumorigenesis and radiation tolerance through PDCD4 ubiquitination. J. Exp. Clin. Cancer Res..

[55] Wang W., Ungermannova D., Chen L., Liu X. (2003). A negatively charged amino acid in Skp2 is required for Skp2-Cks1 interaction and ubiquitination of p27Kip1. J. Biol. Chem..

[56] Jia L., Soengas M.S., Sun Y. (2009). ROC1/RBX1 E3 ubiquitin ligase silencing suppresses tumor cell growth via sequential induction of G2-M arrest, apoptosis, and senescence. Cancer Res..

[57] Abbastabar M., Kheyrollah M., Azizian K., Bagherlou N., Tehrani S.S., Maniati M., Karimian A. (2018). Multiple functions of p27 in cell cycle, apoptosis, epigenetic modification and transcriptional regulation for the control of cell growth: A double-edged sword protein. DNA Repair.

[58] Gorski J.W., Ueland F.R., Kolesar J.M. (2020). CCNE1 Amplification as a Predictive Biomarker of Chemotherapy Resistance in Epithelial Ovarian Cancer. Diagnostics.

[59] Aziz K., Limzerwala J.F., Sturmlechner I., Hurley E., Zhang C., Jeganathan K.B., Nelson G., Bronk S., Fierro Velasco R.O., van Deursen E.J. (2019). Ccne1 Overexpression Causes Chromosome Instability in Liver Cells and Liver Tumor Development in Mice. Gastroenterology.

[60] Dhanasekaran R., Deutzmann A., Mahauad-Fernandez W.D., Hansen A.S., Gouw A.M., Felsher D.W. (2021). The MYC oncogene—The grand orchestrator of cancer growth and immune evasion. Nat. Rev. Clin. Oncol..

[61] Dong Y., Tu R., Liu H., Qing G. (2020). Regulation of cancer cell metabolism: Oncogenic MYC in the driver’s seat. Signal Transduct. Target. Ther..

[62] Ecker A., Simma O., Hoelbl A., Kenner L., Beug H., Moriggl R., Sexl V. (2009). The dark and the bright side of Stat3: Proto-oncogene and tumor-suppressor. Front. Biosci..

[63] Nesbit C.E., Tersak J.M., Prochownik E.V. (1999). MYC oncogenes and human neoplastic disease. Oncogene.

[64] Orlicky S., Tang X., Willems A., Tyers M., Sicheri F. (2003). Structural basis for phosphodependent substrate selection and orientation by the SCFCdc4 ubiquitin ligase. Cell.

[65] Postow L., Funabiki H. (2013). An SCF complex containing Fbxl12 mediates DNA damage-induced Ku80 ubiquitylation. Cell Cycle.

[66] Etemadmoghadam D., Weir B.A., Au-Yeung G., Alsop K., Mitchell G., George J., Australian Ovarian Cancer Study G., Davis S., D’Andrea A.D., Simpson K. (2013). Synthetic lethality between CCNE1 amplification and loss of BRCA1. Proc. Natl. Acad. Sci. USA.

[67] Karst A.M., Jones P.M., Vena N., Ligon A.H., Liu J.F., Hirsch M.S., Etemadmoghadam D., Bowtell D.D., Drapkin R. (2014). Cyclin E1 deregulation occurs early in secretory cell transformation to promote formation of fallopian tube-derived high-grade serous ovarian cancers. Cancer Res..

[68] Rajagopalan H., Jallepalli P.V., Rago C., Velculescu V.E., Kinzler K.W., Vogelstein B., Lengauer C. (2004). Inactivation of hCDC4 can cause chromosomal instability. Nature.

[69] Nakayama K., Nagahama H., Minamishima Y.A., Miyake S., Ishida N., Hatakeyama S., Kitagawa M., Iemura S., Natsume T., Nakayama K.I. (2004). Skp2-mediated degradation of p27 regulates progression into mitosis. Dev. Cell.

[70] Hanahan D., Weinberg R.A. (2011). Hallmarks of cancer: The next generation. Cell.

[71] Nowak M.A., Komarova N.L., Sengupta A., Jallepalli P.V., Shih Ie M., Vogelstein B., Lengauer C. (2002). The role of chromosomal instability in tumor initiation. Proc. Natl. Acad. Sci. USA.

[72] Vishwakarma R., McManus K.J. (2020). Chromosome Instability; Implications in Cancer Development, Progression, and Clinical Outcomes. Cancers.

[73] Gao C., Su Y., Koeman J., Haak E., Dykema K., Essenberg C., Hudson E., Petillo D., Khoo S.K., Vande Woude G.F. (2016). Chromosome instability drives phenotypic switching to metastasis. Proc. Natl. Acad. Sci. USA.

[74] Lee A.J., Endesfelder D., Rowan A.J., Walther A., Birkbak N.J., Futreal P.A., Downward J., Szallasi Z., Tomlinson I.P., Howell M. (2011). Chromosomal instability confers intrinsic multidrug resistance. Cancer Res..

[75] Carter S.L., Eklund A.C., Kohane I.S., Harris L.N., Szallasi Z. (2006). A signature of chromosomal instability inferred from gene expression profiles predicts clinical outcome in multiple human cancers. Nat. Genet..

[76] Lepage C.C., Morden C.R., Palmer M.C.L., Nachtigal M.W., McManus K.J. (2019). Detecting Chromosome Instability in Cancer: Approaches to Resolve Cell-to-Cell Heterogeneity. Cancers.

[77] Thompson L.L., McManus K.J. (2015). A novel multiplexed, image-based approach to detect phenotypes that underlie chromosome instability in human cells. PLoS ONE.

[78] Thompson L.L., Jeusset L.M., Lepage C.C., McManus K.J. (2017). Evolving Therapeutic Strategies to Exploit Chromosome Instability in Cancer. Cancers.

[79] Wang W., Qiu J., Liu Z., Zeng Y., Fan J., Liu Y., Guo Y. (2013). Overexpression of RING box protein-1 (RBX1) associated with poor prognosis of non-muscle-invasive bladder transitional cell carcinoma. J. Surg. Oncol..

[80] Kunishige T., Migita K., Matsumoto S., Wakatsuki K., Nakade H., Miyao S., Kuniyasu H., Sho M. (2020). Ring box protein-1 is associated with a poor prognosis and tumor progression in esophageal cancer. Oncol. Lett..

[81] Yang D., Zhao Y., Liu J., Sun Y., Jia L. (2012). Protective autophagy induced by RBX1/ROC1 knockdown or CRL inactivation via modulating the DEPTOR-MTOR axis. Autophagy.

[82] Liu Y.Q., Wang X.L., Cheng X., Lu Y.Z., Wang G.Z., Li X.C., Zhang J., Wen Z.S., Huang Z.L., Gao Q.L. (2015). Skp1 in lung cancer: Clinical significance and therapeutic efficacy of its small molecule inhibitors. Oncotarget.

[83] Tian C., Lang T., Qiu J., Han K., Zhou L., Min D., Zhang Z., Qi D. (2020). SKP1 promotes YAP-mediated colorectal cancer stemness via suppressing RASSF1. Cancer Cell. Int..

[84] Mao S.Y., Xiong D.B., Huang T.B., Zheng J.H., Yao X.D. (2017). Expression of CUL1 correlates with tumour-grade and recurrence in urothelial carcinoma. ANZ J. Surg..

[85] Chen G., Li G. (2010). Increased Cul1 expression promotes melanoma cell proliferation through regulating p27 expression. Int. J. Oncol..

[86] Bai J., Zhou Y., Chen G., Zeng J., Ding J., Tan Y., Zhou J., Li G. (2011). Overexpression of Cullin1 is associated with poor prognosis of patients with gastric cancer. Hum. Pathol..

[87] Hershko D.D. (2008). Oncogenic properties and prognostic implications of the ubiquitin ligase Skp2 in cancer. Cancer.

[88] Asmamaw M.D., Liu Y., Zheng Y.C., Shi X.J., Liu H.M. (2020). Skp2 in the ubiquitin-proteasome system: A comprehensive review. Med. Res. Rev..

[89] Cai Z., Moten A., Peng D., Hsu C.C., Pan B.S., Manne R., Li H.Y., Lin H.K. (2020). The Skp2 Pathway: A Critical Target for Cancer Therapy. Semin. Cancer Biol..

[90] Petersen I., Kotb W.F., Friedrich K.H., Schluns K., Bocking A., Dietel M. (2009). Core classification of lung cancer: Correlating nuclear size and mitoses with ploidy and clinicopathological parameters. Lung Cancer.

[91] Zeimet A.G., Fiegl H., Goebel G., Kopp F., Allasia C., Reimer D., Steppan I., Mueller-Holzner E., Ehrlich M., Marth C. (2011). DNA ploidy, nuclear size, proliferation index and DNA-hypomethylation in ovarian cancer. Gynecol. Oncol..

[92] Geigl J.B., Obenauf A.C., Schwarzbraun T., Speicher M.R. (2008). Defining ‘chromosomal instability’. Trends Genet..

[93] Barber T.D., McManus K., Yuen K.W., Reis M., Parmigiani G., Shen D., Barrett I., Nouhi Y., Spencer F., Markowitz S. (2008). Chromatid cohesion defects may underlie chromosome instability in human colorectal cancers. Proc. Natl. Acad. Sci. USA.

[94] Galindo-Moreno M., Giraldez S., Limon-Mortes M.C., Belmonte-Fernandez A., Reed S.I., Saez C., Japon M.A., Tortolero M., Romero F. (2019). SCF(FBXW7)-mediated degradation of p53 promotes cell recovery after UV-induced DNA damage. FASEB J..

[95] Orr B., Compton D.A. (2013). A double-edged sword: How oncogenes and tumor suppressor genes can contribute to chromosomal instability. Front. Oncol..

[96] Vaidyanathan S., Cato K., Tang L., Pavey S., Haass N.K., Gabrielli B.G., Duijf P.H. (2016). In vivo overexpression of Emi1 promotes chromosome instability and tumorigenesis. Oncogene.

[97] Erlanson M., Landberg G. (2001). Prognostic implications of p27 and cyclin E protein contents in malignant lymphomas. Leuk. Lymphoma.

[98] Takada M., Zhang W., Suzuki A., Kuroda T.S., Yu Z., Inuzuka H., Gao D., Wan L., Zhuang M., Hu L. (2017). FBW7 Loss Promotes Chromosomal Instability and Tumorigenesis via Cyclin E1/CDK2-Mediated Phosphorylation of CENP-A. Cancer Res..

[99] Cisyk A.L., Penner-Goeke S., Lichtensztejn Z., Nugent Z., Wightman R.H., Singh H., McManus K.J. (2015). Characterizing the prevalence of chromosome instability in interval colorectal cancer. Neoplasia.

[100] Penner-Goeke S., Lichtensztejn Z., Neufeld M., Ali J.L., Altman A.D., Nachtigal M.W., McManus K.J. (2017). The temporal dynamics of chromosome instability in ovarian cancer cell lines and primary patient samples. PLoS Genet..

[101] Morden C.R., Farrell A.C., Sliwowski M., Lichtensztejn Z., Altman A.D., Nachtigal M.W., McManus K.J. (2021). Chromosome instability is prevalent and dynamic in high-grade serous ovarian cancer patient samples. Gynecol. Oncol..

[102] Bonifácio V.D.B. (2020). Ovarian Cancer Biomarkers: Moving Forward in Early Detection. Adv. Exp. Med. Biol..

[103] Moore J.S., Aulet T.H. (2017). Colorectal Cancer Screening. Surg. Clin. N. Am..

[104] Vaser R., Adusumalli S., Leng S.N., Sikic M., Ng P.C. (2016). SIFT missense predictions for genomes. Nat. Protoc..

[105] Adzhubei I.A., Schmidt S., Peshkin L., Ramensky V.E., Gerasimova A., Bork P., Kondrashov A.S., Sunyaev S.R. (2010). A method and server for predicting damaging missense mutations. Nat. Methods.

[106] Zheng N., Schulman B.A., Song L., Miller J.J., Jeffrey P.D., Wang P., Chu C., Koepp D.M., Elledge S.J., Pagano M. (2002). Structure of the Cul1-Rbx1-Skp1-F boxSkp2 SCF ubiquitin ligase complex. Nature.

[107] Hao B., Oehlmann S., Sowa M.E., Harper J.W., Pavletich N.P. (2007). Structure of a Fbw7-Skp1-Cyclin E Complex: Multisite-Phosphorylated Substrate Recognition by SCF Ubiquitin Ligases. Mol. Cell.

[108] Weinhold L., Wahl S., Pechlivanis S., Hoffmann P., Schmid M. (2016). A statistical model for the analysis of beta values in DNA methylation studies. BMC Bioinform..

